# Evaluation of quality of life and physical activity in patients with type 1 diabetes mellitus during the COVID-19 pandemic

**DOI:** 10.20945/2359-3997000000595

**Published:** 2023-01-25

DOI: 10.20945/2359-3997000000531

Ahead of print

Where you read:

**ABSTRACT**

**Objective:** The aim of the study is to compare the quality of life, physical activity, anxiety, depression, fear of hypoglycemia, loneliness perception in patients with type 1 diabetes mellitus and controls. **Subjects and methods:** Forty-four patients and 63 controls were included in this cross-sectional study. Quality of life (Short Form 36-SF-36), physical activity level (International Physical Activity Questionnaire-short form), anxiety and depression (Hospital Anxiety and Depression Scale), fear of hypoglycemia (Hypoglycemia Fear Survey), loneliness perception (UCLA Loneliness Scale) were evaluated. **Results:** Physical role limitations and general health perception subscale scores of SF-36 questionnaire in patients were **significantly higher** than the controls (p < 0.05). **Conclusion:** Role limitations due to physical problems and fear of hypoglycemia are increased, and general health perception is impaired in patients with type 1 diabetes mellitus. Physical inactivity is an important symptom in individuals in the pandemic period. In this regard, telerehabilitation approaches will be beneficial for all individuals in increasing physical activity, improving quality of life, and decreasing anxiety, depression and loneliness perception during the pandemic period for all individuals. The importance of a multidisciplinary approach in diabetes management and dealing with problems should be considered in pandemic.

Should read:

**ABSTRACT**

**Objective:** The aim of the study is to compare the quality of life, physical activity, anxiety, depression, fear of hypoglycemia, loneliness perception in patients with type 1 diabetes mellitus and controls. **Subjects and methods:** Forty-four patients and 63 controls were included in this cross-sectional study. Quality of life (Short Form 36-SF-36), physical activity level (International Physical Activity Questionnaire-short form), anxiety and depression (Hospital Anxiety and Depression Scale), fear of hypoglycemia (Hypoglycemia Fear Survey), loneliness perception (UCLA Loneliness Scale) were evaluated. **Results:** Physical role limitations and general health perception subscale scores of SF-36 questionnaire in patients were **significantly lower** than the controls (p < 0.05). **Conclusion:** Role limitations due to physical problems and fear of hypoglycemia are increased, and general health perception is impaired in patients with type 1 diabetes mellitus. Physical inactivity is an important symptom in individuals in the pandemic period. In this regard, telerehabilitation approaches will be beneficial for all individuals in increasing physical activity, improving quality of life, and decreasing anxiety, depression and loneliness perception during the pandemic period for all individuals. The importance of a multidisciplinary approach in diabetes management and dealing with problems should be considered in pandemic.

Where you read:

**RESULTS**

A total of 118 individuals replied to the online survey. Finally, 44 patients (27.5 ± 7.12 years) and 63 controls (27.65 ± 7.16 years) were selected for analysis (Figure 1). Demographic characteristics were similar except for smoking (p < 0.05). The demographic and clinical characteristics of patients were shown in Table 1. Patients were all on intensive insulin therapy using multiple daily injections of 3 rapid acting and 1 long acting insulin or continuous subcutaneous insulin infusion with rapid acting insulin. Physical role limitations and general health perception subscale scores of SF-36 questionnaire in patients were statistically **significantly higher** compared with controls (Table 2, p < 0.05). Other parameters were similar in both groups (p > 0.05). Physical activity levels of participants were similar (Table 2, p > 0.05). However, 12 (28%) patients and 18 (29%) controls had low, 24 (56%) patients and 31 (50%) controls had moderate, 7 (16%) patients and 13 (21%) controls had high physical activity levels. Fifty (79.4%) controls and 29 (65.9%) patients were had moderate physical activity for less than 150 min/week. Fifty (79.4%) controls and 33 (75%) patients were had vigorous physical activity for less than 75 min/week. Anxiety and depression scores were similar in both groups (Table 2, p > 0.05). However, 20.5% of patients and 15.9% of controls had anxiety; 34.1% of patients and 34.9% of controls had depression. Eight (18.2%) patients’ subscale score of HFS-B, 7 (15.9%) patients’ subscale score of HFS-W and 9 (20.5%) patients’ total score of HFS was above 50%. The total score of HFS in patients was 28.32 ± 15.59 (Table 1). ULS-8 scale scores were similar in both groups (Table 2, p > 0.05). The HbA1c levels of 65.1% of patients were above 7 mmol/mol.

Should read:

**RESULTS**

A total of 118 individuals replied to the online survey. Finally, 44 patients (27.5 ± 7.12 years) and 63 controls (27.65 ± 7.16 years) were selected for analysis (Figure 1). Demographic characteristics were similar except for smoking (p < 0.05). The demographic and clinical characteristics of patients were shown in Table 1. Patients were all on intensive insulin therapy using multiple daily injections of 3 rapid acting and 1 long acting insulin or continuous subcutaneous insulin infusion with rapid acting insulin. Physical role limitations and general health perception subscale scores of SF-36 questionnaire in patients were statistically **significantly lower** compared with controls (Table 2, p < 0.05). Other parameters were similar in both groups (p > 0.05). Physical activity levels of participants were similar (Table 2, p > 0.05). However, 12 (28%) patients and 18 (29%) controls had low, 24 (56%) patients and 31 (50%) controls had moderate, 7 (16%) patients and 13 (21%) controls had high physical activity levels. Fifty (79.4%) controls and 29 (65.9%) patients were had moderate physical activity for less than 150 min/week. Fifty (79.4%) controls and 33 (75%) patients were had vigorous physical activity for less than 75 min/week. Anxiety and depression scores were similar in both groups (Table 2, p > 0.05). However, 20.5% of patients and 15.9% of controls had anxiety; 34.1% of patients and 34.9% of controls had depression. Eight (18.2%) patients’ subscale score of HFS-B, 7 (15.9%) patients’ subscale score of HFS-W and 9 (20.5%) patients’ total score of HFS was above 50%. The total score of HFS in patients was 28.32 ± 15.59 (Table 1). ULS-8 scale scores were similar in both groups (Table 2, p > 0.05). The HbA1c levels of 65.1% of patients were above 7 mmol/mol.

